# Protocol for the standardized design of Morris water maze experiments and statistical analysis using SPSS

**DOI:** 10.1016/j.xpro.2026.104509

**Published:** 2026-04-29

**Authors:** Xiao-Yun Hu, Meng-Jie Xu, Xiang-Hu Liu, Tian Xia, Qi-Gang Zhou, Jian-Dong Jiang, Jing Zhang

**Affiliations:** 1 Department of Clinical Pharmacology, School of Pharmacy, Nanjing Medical University, Nanjing, Jiangsu 210029, China; 2 Department of Pharmacy of First Affiliated Hospital of Nanjing Medical University, Nanjing 210029, China

**Keywords:** Neuroscience, cognitive Neuroscience, Behavior

## Abstract

The Morris water maze is a widely used paradigm for assessing spatial learning and memory in rodents; however, variability in procedures and analysis can lead to inconsistent results across laboratories. Here, we present a protocol for the standardized design of Morris water maze experiments and statistical analysis using SPSS. We describe procedures for preliminary screening of mice, spatial data acquisition, assessment of spatial working memory and reference memory, as well as detailed SPSS operation and result interpretation.

## Highlights

• Steps for conducting Morris water maze experiments
• Criteria for excluding abnormal mice before Morris water maze testing
• Step-by-step SPSS procedures for outlier removal and statistical analysis of maze data

## Abstract (editor highlights)

Publisher’s note: Undertaking any experimental protocol requires adherence to local institutional guidelines for laboratory safety and ethics.

## Graphical abstract

**Figure:**
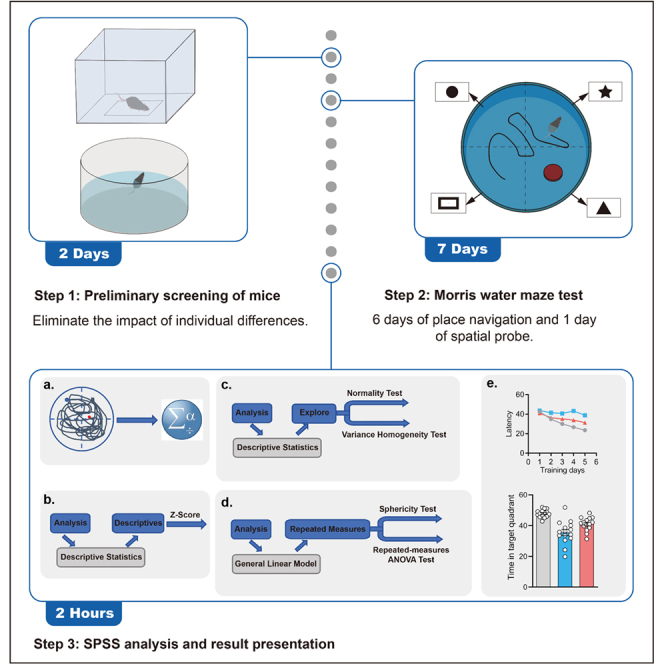

## Before you begin

The Morris Water Maze (MWM) is widely used to assess hippocampus-dependent spatial learning and reference memory in rodents.^1^^,^^2^ Since the 1980s, it has become a cornerstone assay in neuroscience, behavioral pharmacology, and neuropsychology, used in studies of neurodegenerative diseases, developmental disorders, and antidepressant mechanisms.^3^^,^^4^ The strength of the MWM lies in its reliability across species and laboratories, its independence from food or water restriction, and its ability to capture spatial information during probe trials.^5^

Despite its ubiquity, MWM data analysis remains inconsistent.^6^^,^^7^ Many studies use simple t-tests at individual time points, neglecting the repeated-measures structure of acquisition data and within-subject correlations.^8^^,^^9^^,^^10^ Such approaches increase variability, reduce statistical power, and complicate cross-study comparisons. Probe data analysis also varies, with no clear justification or multiple comparison corrections.

Here, we provide a standardized, reproducible IBM SPSS statistical protocol for MWM acquisition and probe data. Acquisition data (e.g., escape latency, path length) are analyzed with repeated-measures analysis of variance (RM-ANOVA) or multivariate analysis of variance (MANOVA), including assumption testing, sphericity corrections, and post hoc comparisons. Probe outcomes are analyzed with group-level comparisons using parametric or non-parametric methods as appropriate. This protocol is designed to be accessible to researchers with limited statistical background while maintaining rigor and transparency.

### Innovation

In the water maze learning experiment, the performance of the animals usually changes over time (for example, the latency period gradually shortens), resulting in uneven variances between different time points. The spherical test can effectively identify the violation of statistical assumptions caused by this learning curve, thereby selecting an analysis method that is more in line with the characteristics of the behavioral data. In this article, we employ techniques such as normality analysis, homogeneity of variance test, and sphericity test to ensure the accuracy of the statistical results. This is an important step in the data processing procedure, assisting researchers in making more scientific and reliable method choices when analyzing complex animal behavior data.

### Institutional permissions

All animal procedures should comply with institutional and national guidelines. The data used in this analysis were collected under approval from the Institutional Animal Care and Use Committee (IACUC) of Nanjing Medical University. No additional permissions are required for statistical analysis using SPSS.

## Key resources table

**Table:** 

REAGENT or RESOURCE	SOURCE	IDENTIFIER
**Experimental models: Organisms/strains**		

C57BL6/J male mice from 8 to 12 weeks	Institutional Animal Care and Use Committee (IACUC) of Nanjing Medical University (NJMU)	N/A
Tert^−/−^ male mice from 8 to 12 weeks	Laboratory-constructed, housed in Institutional Animal Care and Use Committee (IACUC) of Nanjing Medical University (NJMU)	N/A

**Software and algorithms**		

IBM SPSS Statistics 27	IBM	https://www.ibm.com/cn-zh/products/spss
Smart 3.0	Panlab	https://www.panlab.com/en/
Camera	Lenovo	https://www.lenovo.com.cn
Excel	Microsoft	https://www.microsoft.com/

**Other**		

MWM pool	RWD,Shenzhen	https://www.rwdls.com/
Camera	Xiaomi	Cat# BW500
Liquid nontoxic white paint	Maries	N/A
Red flag	Stationery shop	N/A
Thicken latex gloves	Stationery shop	N/A

## Materials and equipment

**Table:** 10mg/kg Ketamine solution

Reagent	Final concentration	Amount
Ketamine solution (25mg/ml)	25 mg/ml	200 uL
0.9% NaCl solution	0.9 mg/mL	4.8 mL
**Total**	N/A	5 mL

[Store at −4°C for up to 3 days].

**CRITICAL:** Ketamine is a controlled substance and must be stored in a locked container. Waste liquid and discarded ketamine solution shall be disposed of in accordance with the laboratory hazardous waste regulations and must not be dumped at will.

## Step-by-step method details

### Preliminary screening of mice for water maze tests

**Timing: 5 days (for steps 1 and 2)**

**Timing: 2 days (for steps 3–5)**

Here, we describe steps for preliminary screening and preparation of mice before MWM testing.

***Note:*** Mice used for the MWM should undergo preliminary screening of motor and swimming ability before the experiment to minimize individual variability and prevent basic ability differences from affecting subsequent experimental results.

1. Select adult male mice of similar ages and weights (8–12 weeks, 20–25 g), and randomly group them according to experimental designs.
2. Acclimate mice to gentle physical contact for 3 min daily prior to experimental initiation to minimize stress responses to subsequent experimental manipulations.
3. Perform an open-field test (OFT) to preliminarily assess basic locomotor activity using the total distance traveled as an index, and exclude animals with outliers.

**CRITICAL:** Mice with higher locomotor motor ability usually swim faster and reach the platform sooner, which may lead to false-positive results in MWM performance.

4. Place each mouse in the pool without any visual cues or platform. Record the total swimming distance within 1 minute to assess swimming ability, and exclude mice with outliers.

**CRITICAL:** Mice showing excessive stress or immobility in water may produce false-positive or false-negative results.

5. After screening, confirm that there are no significant differences in baseline performance among groups before initiating MWM testing (Bayesian factor is not statistically significant, *p > 0.001*).

### Spatial data acquisition

**Timing: 1 day**

Here, we describe steps for spatial data acquisition in the Morris Water Maze test.

6. Attach numbered ear tags to each mouse at three weeks of age to ensure individual identification throughout the experiment.
7. Divide the water tank (120 cm in diameter and 60 cm in height) into four quadrants, and at the center of the water tank wall in each quadrant, attach a unique pattern (high-contrast, large-sized geometric shapes).
8. Place the platform in the fourth quadrant (Figure 1B).

**Figure 1:**
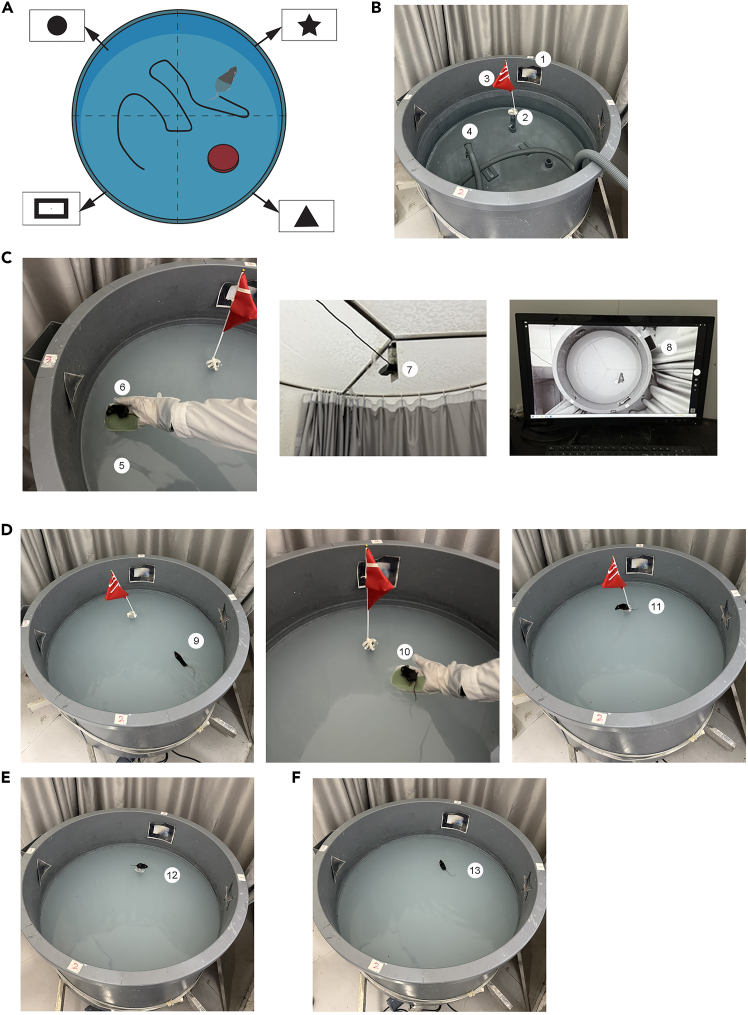
Standardized setup and behavioral procedure for the Morris water maze test (A) Schematic illustration of the Morris water maze showing the circular pool, hidden platform location (red circle), and four fixed distal visual cues (black circle, star, triangle, and rectangle) placed on the surrounding curtain. The dotted line represents a representative swim path during training. (B) Overview of the MWM apparatus including (1) external visual cues, (2) submerged escape platform, (3) flag marker for platform identification during the visible trial, and (4) water circulation system for maintaining uniform temperature. (C) Setup and calibration of the water maze: (5) opaque water maintained at 20–22°C, (6) experimenter placing the hidden platform, (7) ceiling-mounted video camera for tracking, and (8) computer-based tracking interface for real-time monitoring. (D) Acquisition phase: (9) mouse released from a random start position facing the pool wall, (10) guiding the mouse to the platform after 60 s if unsuccessful, and (11) mouse resting on the platform for 15–20 s between trials. (E) Probe trial: (12) platform removed and mouse swimming freely for 60 s to assess spatial memory retention. (F) Representative search behavior: (13) mouse performing target-oriented search, indicating successful learning of platform location.
9. Place a small flag on the platform to attract the attention of the animals (Figure 1B).
10. Gently place the mouse at the designated start position, facing the visual cue on the pool wall (Figure 1C).

**CRITICAL:** When putting the animal into the water, do so gently to avoid stress (do not throw it directly into the water).

**CRITICAL:** When the water temperature is below 20°C, it is necessary to use an integrated heating system to adjust the water temperature to 20–22°C. This is to prevent the mice from experiencing any adverse reactions due to the low water temperature.

**CRITICAL:** To make the pictures clear, we took the pictures in a light environment. However, in the actual MWM test. The experiment should be conducted in an environment with indirect lighting and constant illumination intensity.

11. Start the stopwatch and the computer tracking program (Smart3.0) as soon as the animal is placed in the water (Figure 1C).
12. Stop the stopwatch when the animal boarded the platform and stayed there.

***Note:*** Most animals will climb onto the platform, but there are exceptions. If the animals fail to find the platform within the time limit, they will be guided to it (Figure 1D).

***Note:*** The standard trial time limit for each trial is 1–2 mins.

13. Keep the experimental animals on the platform for 15 s to enhance the learning outcome (Figure 1D).

**CRITICAL:** For mice, longer test intervals (especially during the first test) are usually employed, which can improve the learning effect. The purpose of keeping the animals on the platform is to allow them to adapt to their position in the space and remember the location of the target relative to the surrounding cues.

14. Place the animal at a new starting position in the maze, and then repeat the trials (steps 10–13) until the animal has completed the required number of trials for the day.

***Note:*** Under normal circumstances, animals undergo multiple tests every day. The most common frequency is three times. As there are four quadrants, except for the fourth quadrant where the platform is placed, the animals are tested in the other three quadrants. Each animal is tested three times a day.

**CRITICAL:** Different studies use varying numbers of trials (1–12 per day). No clear advantage has been demonstrated for any particular schedule. Some evidence suggests fewer trials per day may enhance learning, though findings are inconsistent and may depend on pool size.

### Spatial working memory

**Timing: 5 days**

Here, we describe steps for assessing spatial working memory in the Morris Water Maze.

15. Remove the flag from the fourth quadrant platform.
16. Place the animals at random desired starting positions in the maze (quadrants one, two, and three) with their heads facing the pattern on the tank wall.

**CRITICAL:** Keep the animal in the water gently to prevent stress (do not throw directly into the water).

17. Activate the stopwatch and computer tracking program upon placing the animal in the water.
18. Stop the stopwatch when the animal gets on the platform. The standard trial time limit for each trial is 1 to 2 mins.
19. Place the animal in a new starting position in the maze, and the trial is repeated (steps 16–18) until the animal has completed the 3 trials required for that day (Figure 1E).
20. Test the animals in three of the four quadrants, excluding the quadrant containing the platform.
21. Perform three trials per animal per day.

### Reference memory

**Timing: 1 day**

Here, we describe steps for testing reference memory in the Morris Water Maze.

22. Remove the platform in quadrant 4.
23. Place the animal in the second quadrant of the maze (desired starting position) so that it faces the pattern on the wall of the tank.

**CRITICAL:** Keep the animal in the water gently to prevent stress (do not throw directly into the water).

24. Activate the stopwatch and computer tracking program upon placing the animal in the water.
25. Count the time the animals spend in the fourth quadrant (the platform quadrant) within the specified test period (Figure 1F).

### SPSS operation steps

**Timing: 1 h**

Here, we describe steps for statistical analysis of MWM data using SPSS.

**Figure 3:**
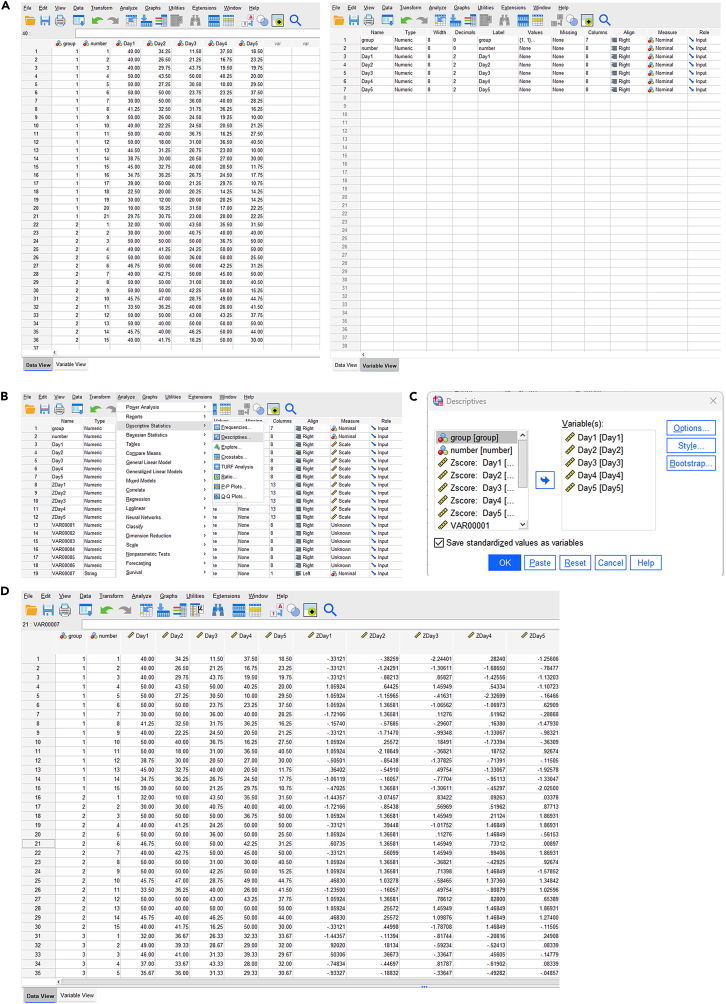
Data preprocessing and outlier screening of Morris water maze data in SPSS (A) Variable definition interface in Variable View. (B) Menu path for descriptive statistics (Analyze > Descriptive Statistics > Descriptives). (C) Selection of Day1–Day5 variables and setting of *Save standardized values as variables*. (D) Screening and exclusion of outliers with absolute *Z*-score ≥ 3 for *Z*Day1–*Z*Day5.

26. Input data (Figure 2A)
  a. Open the window for original data from our behavioral test (Data S1) input by clicking ‘Data View’ button in SPSS software.
  b. Define the variable parameter in ‘Variable View’ (Figure 3A).

**Figure 2:**
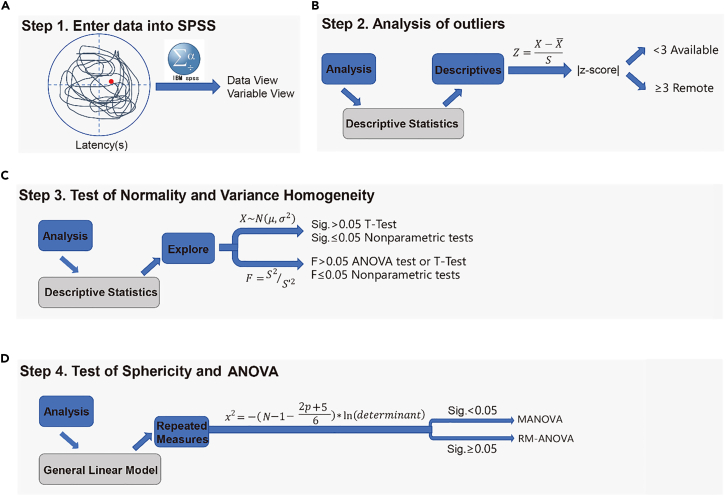
Analysis steps of SPSS (A) Input data into the data view module of SPSS and define Variable parameters in the Variable view module. (B) Outlier values of data obtained by SPSS analysis, and elimination of outlier values can reduce the probability of misjudgment to a certain extent. (C) Normal distribution test and homogeneity test were performed by SPSS analysis. (D) Spherical test and RM-ANOVA were performed by SPSS analysis.
27. Analysis of outliers (Figure 2B).
  a. Open ‘Analyze > descriptive statistics > Descriptives’ (Figure 3B).
  b. Select ‘Day1-Day5′ in the ‘Variable(s)' box, select ‘Save standardized values as variables’, and click ‘OK’ (Figure 3C).
  c. Filter ‘ZDay1-ZDay5′, and exclude the data with absolute value greater than or equal to 3 (Figure 3D).

DESCRIPTIVES VARIABLES=Day1 Day2 Day3 Day4 Day5

 /SAVE

 /STATISTICS=MEAN STDDEV MIN MAX.

**Figure 4:**
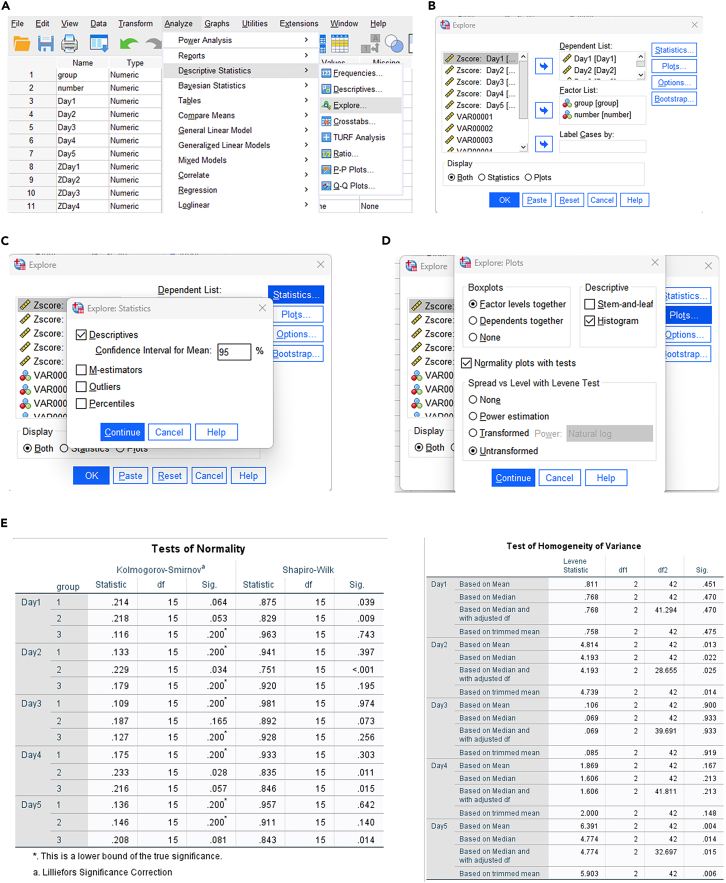
Normality test and variance homogeneity analysis of Morris water maze data in SPSS (A) Menu path for Explore procedure (Analyze > Descriptive Statistics > Explore). (B) Setting of dependent variables (Day1–Day5) and factor variable (*group* and *number*). (C) Statistics setting: Descriptives with 95% confidence interval for the mean. (D) Plots setting: Factor level together, Histogram, and Normality plots with tests. (E) Output results of normality test and variance homogeneity test.

28. Test of Normality and Variance Homogeneity (Figure 2C).
  a. Open ‘Analyze > descriptive statistics > Explore’ (Figure 4A).

**Figure 5:**
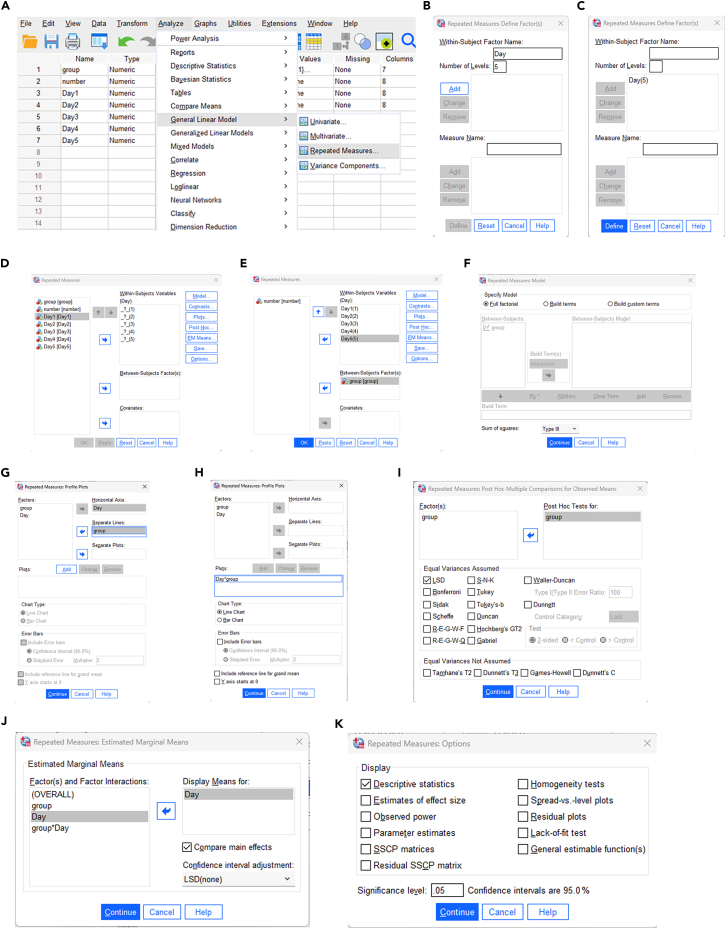
Repeated-measures ANOVA and sphericity test of Morris water maze data in SPSS (A) Menu path for repeated-measures ANOVA (Analyze > General Linear Model > Repeated Measures). (B) Definition of within-subject factor: factor renamed as *Day*, with 5 levels. (C) Interface for defining within-subjects variables after factor establishment. (D) Assignment of within-subjects variables (Day1–Day5). (E) Assignment of between-subjects factor (*group*). (F) Model setting: Type III sum of squares for balanced data (Type IV for unbalanced data). (G) Profile plot setting: *Day* as horizontal axis, *group* as separate lines. (H) Confirmation of *Day*∗*group* interaction plot. (I) Post hoc test setting: LSD for pairwise comparisons between groups. (J) Options setting: Display means for *Day* and Compare main effects. (K) Final output of descriptive statistics, sphericity test, and RM-ANOVA results.
  b. Select ‘Day1-Day5′ in the ‘Dependent List’ box, and then select ‘group’ and ‘number’ in the ‘Factor List’ box (Figure 4B).
  c. Click the ‘Statistics’ button in the upper right corner, select ‘Descriptives’ and set the ‘Confidence Interval for Mean’ to 95% (Figure 4C).
  d. Click ‘Continue’ to return.
    i. Click the ‘Plots’ button, and enter the following dialog box (Figure 4D).
    ii. Select the check box of ‘Factor level together’ and ‘Histogram’, and select ‘Normality plots with tests’ (Figure 4D).
    iii. Click ‘Continue’ to return, and click ‘OK’ to output the result (Figure 4D).

EXAMINE VARIABLES=Day1 Day2 Day3 Day4 Day5 BY group number

/PLOT BOXPLOT HISTOGRAM NPPLOT SPREADLEVEL(1)

/COMPARE GROUPS

/STATISTICS DESCRIPTIVES

/CINTERVAL 95

/MISSING LISTWISE

/NOTOTAL.

29. Test of Sphericity and RM-ANOVA (Figure 2D).
  a. Open ‘Analyze > General Linear Model > Repeated measures’ (Figure 5A).
  b. The Repeated Measures Define Factor(s) dialog box appears.
    i. Within Subject Factor Name: Change ‘factor1′ to ‘Day’ (Figure 5B).
    ii. Because the subject has been tested for a total of 5 days, and Enter 5 in the ‘Number of Levels’ field (Figure 5B).
  c. Click the ‘Add’ button, and the ‘Define’ button below becomes available. Click to enter the following dialog box (Figure 5C).
  d. Select ‘Day1-Day5′ into the ‘Within-Subjects Variables’ box (Figure 5D).
  e. Select ‘group’ into the ‘Between-Subjects Factor(s)' box (Figure 5E).
  f. Click the ‘Model’ button in the upper right corner to output the main effect of the treatment factor and time, as well as the results of the interaction effect test (Figure 5E). ‘Sum of squares’ selects ‘Type III’ (Figure 5F).
    ***Note:*** This applies to balanced data, that is, the number of samples in each group is the same. For non-equilibrium data, select Type IV.
  g. Click ‘Continue’ to return and click the ‘Plots’ button.
    i. Enter the following dialog box: select ‘Horizontal Axis’ for Day, select ‘Separate Lines’ for group (Figure 5G).
    ii. Click the ‘Add’ button (Figure 5G).
    iii. Display ‘Day∗group’ in the box below (Figure 5H).
  h. Click ‘Continue’ to return, click the ‘Post Hoc’ button.
    i. Select group in the right ‘Post Hoc Tests for’ box (Figure 5I).
    ii. Check the box ‘LSD’, and conduct pairwise comparison between groups (Figure 5I).
  i. Click ‘Continue’ to return, click the ‘Options’ button.
    i. Enter the following dialog box: select ‘day’ into ‘Display Means for’.
    ii. Check the box of ‘Compare main effects’ (Figure 5J).
    iii. Check the box of ‘Descriptive Statistics’ below (Figure 5K).
    iv. Set the significance level to 0.05 (Figure 5K).
  j. Click ‘Continue’ to return, and click ‘OK’ to output the result (Figure 5K).
    GLM Day1 Day2 Day3 Day4 Day5 BY group
     /WSFACTOR=Day 5 Polynomial
     /METHOD=SSTYPE(3)
     /POSTHOC=group(LSD)
     /PLOT=PROFILE(Day∗group) TYPE=LINE ERRORBAR=NO MEANREFERENCE=NO YAXIS=AUTO
     /EMMEANS=TABLES(Day) COMPARE ADJ(LSD)
     /PRINT=DESCRIPTIVE
     /CRITERIA=ALPHA(.05)
     /WSDESIGN=Day
     /DESIGN=group.

### Analysis of the result

**Timing: 1 h**

Here, we describe steps for interpreting, visualizing and presenting MWM experimental results.

30. Output overview.
  ***Note:*** After running the RM-ANOVA, SPSS automatically generates multiple out-put tables summarizing within- and between-subject effects, assumption test-s, and post hoc results.
  a. The first table (Within-Subjects Factors) confirms the definition of the repeated-measures structure, listing the five training days as within-subject levels and the experimental group as the between-subjects factor (Figure S1A).
31. Assumption testing.
  a. Check Mauchly’s Test of Sphericity (Figure S1B) to evaluate whether the assumption of sphericity is satisfied.
  b. If the test is not significant (*p > 0.05*), meaning ‘Sphericity Assumed’ results, apply the repeated-measures ANOVA(Figure 2D).
  c. If significant (*p < 0.05*), apply the Greenhouse–Geisser or Huynh–Feldt correction to adjust degrees of freedom.
    ***Note:*** Alternatively, multivariate analysis of variance (MANOVA) or linear mixed models (LMM) were used instead to avoid assumption restrictions.
  d. To validate robustness, refer to Multivariate Tests (Pillai’s Trace, Wilks’ Lambda, Hotelling’s Trace, Roy’s Root), which do not assume sphericity (Figure S1H).
    **CRITICAL:** Since the sphericity test in this data is not significant, which meets the sphericity test requirements, only RM-ANOVA analysis is conducted here, and MANOVA analysis is not performed.
32. Within-subject and between-subject effects.
  a. Examine the Tests of Within-Subjects Effects table (Figure S1C).
    i. The main effect of Day tests overall learning across sessions.
    ii. The Day × Group interaction tests whether learning trajectories differ among groups.
  b. Inspect Tests of Within-Subjects Contrasts (Figure S1D) to determine whether performance changes linearly or non-linearly across training days.
  c. Review the Tests of Between-Subjects Effects (Figure S1E) to identify group-level differences independent of time.
  d. The Estimates table (Figure S1F) reports adjusted marginal means and 95% confidence intervals for each group and condition.
33. Post hoc and multivariate comparisons.
  a. When significant main or interaction effects are found, use Pairwise Comparisons and Multiple Comparisons outputs for detailed post hoc tests (Figure S1G and S1H).
  b. Select the LSD or Bonferroni correction to control for multiple testing.
  c. Asterisks in the output indicate comparisons reaching statistical significance *(p < 0.05*).
  d. Complementarily, Multivariate Tests (Figure S1I) can be reported to confirm the overall significance of the within-subject factor ‘Day’.
34. Visualization and interpretation.
  a. The Estimated Marginal Means plot (Figure S1J) illustrates group performance across training days.
  b. Decreasing latency across sessions indicates successful learning; a flatter curve or higher overall latency suggests impaired spatial learning.
  c. Report the corrected *F* and *p* values, effect sizes (*η*^*2*^), and observed power for transparency and reproducibility.
  d. Example reporting format:

 “RM-ANOVA revealed a significant main effect of Day (*F*_*(4, 48)*_ *= 15.99, p < 0.001, η*^*2*^ *= 0.59*) and a significant Day × Group interaction (*F*_*(8, 96)*_ *= 4.78, p < 0.01, η*^*2*^ *= 0.28*). Post hoc tests indicated that group 3 showed shorter escape latencies than group 1 from Day 3 onward.”

**Figure 6:**
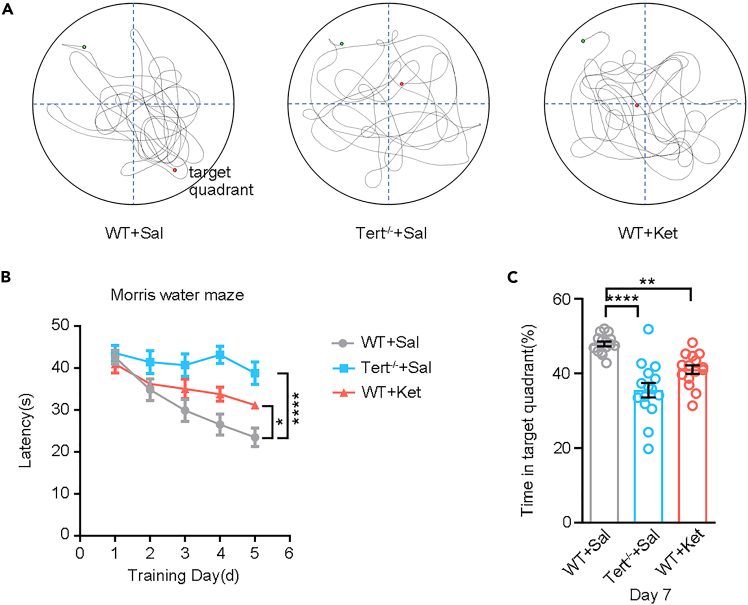
Validation of the standardized Morris water maze protocol in Tert^−/−^and ketamine-treated^11^^,^^12^ mice (A) Representative swim trajectories during the probe trial on Day 7. Wild-type mice treated with normal saline (WT+Sal) showed target-oriented search patterns, whereas Tert^−/−^ + Sal and WT + ketamine mice exhibited disorganized swim paths and reduced preference for the target quadrant. (B) Learning curves across 5 training days. WT+Sal mice displayed progressively reduced escape latency, indicating normal spatial learning, while Tert^−/−^ and ketamine-treated mice showed impaired learning performance. (C) Quantification of probe-trial performance on Day 7. Both Tert^−/−^ and ketamine-treated groups spent significantly less time in the target quadrant compared with WT+Sal controls (*p < 0.01*), demonstrating memory retention deficits.
  b. Evaluate reference memory (Data S2) by recording the time spent in the target quadrant within the allotted period on day 7 (Figure 6C).

35. Result presentation.
  a. Assess spatial working memory (Data S1) using the trajectory plot using the trajectory plots of animal movements in the Morris water maze during the test period (Figure 6A), as well as the line chart of escape latency from day 2 to day 6 (Figure 6B).

## Expected outcomes

The analysis yields learning curves showing decreasing latency across days and possible group differences. The main effect of Day indicates learning; a Day × Group interaction indicates treatment effect. Probe analysis reveals retention differences.

## Limitations

In our study, we conducted a 5-days MWM test on 3 groups of mice and analyzed the effects of factors such as “group” and “days” on the escape latency of the mice.

First, we performed inter-group homogeneity of variance tests on the behavioral data of each day to verify whether the variances of the dependent variables of mice in different groups were homogeneous on each day, so as to prevent significant misjudgment effects caused by unequal variances. Compared with the Bartlett test, we chose the Levene test, which is more robust and more sensitive to non-normal data. However, the Levene test is very sensitive to “extreme values”. Abnormal latency data caused by operational errors in individual mice may directly lead to a Levene test Sig. < 0.05. Although we excluded some abnormal mice through swimming ability tests before the experiment, it is still necessary to detect outliers in the subsequent results. We suggest that outliers can be identified through box plots or *Z*-scores to determine whether they are measurement errors; otherwise, the test efficiency will be reduced.

Meanwhile, for the repeated-measures experimental design like the water maze, the Levene test needs to be analyzed separately by day, which may not be able to overall evaluate the temporal dynamics of variance heterogeneity. This is essentially unavoidable because the Levene test is inherently *static and single-time-point*, which does not match the *dynamic and time-series* characteristics of repeated-measures data. This mismatch not only makes it impossible to explain the changes in variance during the water maze learning process but also increases the complexity of the analysis through *multiple testing risks, contradictory interpretations, and cumbersome operations*. For some more complex experiments, testing the variance structure of the mixed linear model may be necessary.

To determine whether there is a high correlation between the data of 5 repeated measurements, we used the sphericity test to verify whether the variances of differences between different days are equal. Compared with the complex statistics of MANOVA, the calculation logic of the sphericity test is clear and the output results are concise. When sphericity holds, the effect size calculation of RM-ANOVA is more accurate, and it can directly quantify the actual impact of “day effect” and “group effect”. However, when the sample size is too large or too small, the data may show obvious skewness and missing values.

When the sphericity test is not satisfied, MANOVA is an important supplement to the repeated-measures design. It can overall evaluate the interaction effect of “group × days” through multivariate statistics (Wilks’ Lambda), avoiding the fragmentation of single-time-point analysis. At the same time, it can also include multiple behavioral indicators (such as analyzing escape latency and the number of platform crossings simultaneously) to more comprehensively reflect the spatial learning and memory ability of mice. However, MANOVA has high requirements on sample size; otherwise, the Wilks’ Lambda estimation will be biased and the Sig. value will be inaccurate. We suggest that the number of mice should be more than 20 when conducting MANOVA.

In conclusion, among the above analysis methods, we adopted the homogeneity of variance test and Mauchly’s test of sphericity to ensure the validity of the parametric tests. The Levene test can accurately identify variance heterogeneity on different days, avoiding false positives caused by unequal variances; the sphericity test directly addresses the core assumption of repeated-measures data, solving the problem of traditional ANOVA’s dependence on sphericity and improving the reliability of results.

## Troubleshooting

### Problem 1

The “Repeated Measures” option in SPSS is unavailable. Related to step 39a.

### Potential solution

The dataset is not in wide format. Reorganize your data so that each training day is represented by a separate column (e.g., Latency Day1–Latency Day5) and each row corresponds to a single subject. Save the file in.sav format before re-analysis.

### Problem 2

Mauchly’s test of sphericity is significant (p < 0.05). Related to step 31c.

### Potential solution

The assumption of sphericity is violated because repeated measures are correlated. Apply the Greenhouse–Geisser correction to adjust degrees of freedom and report corrected F and p values. Alternatively, perform MANOVA, which does not assume sphericity.

### Problem 3

Some subjects are automatically excluded from the repeated-measures ANOVA.

### Potential solution

Missing values exist for one or more training days. Identify incomplete cases and either remove them or use mixed-effects modeling. Always inspect raw data for blank cells before running ANOVA.

### Problem 4

Levene’s test indicates unequal variances across groups. Related to limitations.

### Potential solution

Variance heterogeneity violates ANOVA assumptions. Use the “Robust Tests of Equality of Means” option in SPSS, or apply data transformation (e.g., log-transform latency). If variance remains unequal, choose nonparametric tests such as the Mann–Whitney U test.

### Problem 5

No significant Day × Group interaction, but group curves appear visually different. Related to step 32a.

### Potential solution

The dataset may have insufficient power or high within-group variability. Increase sample size or run polynomial contrast analyses to examine trend differences. Report effect sizes (*η*^*2*^) even when the interaction term is not significant.

### Problem 6

No significant main effect of Day (no learning observed).

### Potential solution

This may result from data entry errors or a failed learning process. Verify that data are correctly ordered (Day1–Day5) and confirm experimental integrity (consistent cues, water temperature, platform stability). If learning truly did not occur, interpret results cautiously.

### Problem 7

Mice exhibited significant operational stress (struggling, squeaking) despite 3 min of daily pre-experimental acclimation handling. Related to step 2.

### Potential solution

Gradually increase handling duration from 1 min/day to 3 min (only after no struggling); the same experimenter gently stroked the mice’s back (avoid sensitive head/abdomen) with powder-free latex gloves, and performed handling beside housing cages (not the novel experimental room) to reduce cumulative stress.

### Problem 8

After being placed in water, mice swam randomly along the tank edge instead of orienting to the visual patterns on the wall, failing to recognize spatial cues. Related to step 10.

### Potential solution

Use large (≥10cm×10cm), high-contrast black-white geometric patterns (e.g., black squares/white triangles; mice recognize grayscale and contours far better than colors), avoiding thin lines/small patterns; attach patterns to the upper-middle inner tank wall (∼10cm above water) for clear viewing when mice look up, preventing occlusion or out-of-view placement; place mice with heads facing pattern centers, hold for 2s after limbs touch water before release to ensure initial visual focus on spatial cues.

### Problem 9

Mice developed learning fatigue during 3 daily test trials, with a significant prolongation of latency in the last 1–2 trials. Related to step 16.

### Potential solution

After each test, place mice on a 37°C constant-temperature warming pad for 10–15 min of rest for physical recovery before the next test; randomly select 3 entry points in non-target quadrants for the 3 tests to avoid the formation of path memory (instead of spatial memory) caused by fixed entry points.

## Resource availability

### Lead contact

Further information and requests for resources and reagents should be directed to and will be fulfilled by the lead contact, Jing Zhang (zj1984@njmu.edu.cn).

### Technical contact

Questions about the technical specifics related to performing the protocol should be directed to and will be answered by the technical contact, Xiao-Yun Hu (hxy0906@stu.njmu.edu.cn).

### Materials availability

This study did not generate new unique materials.

### Data and code availability

All raw data used in this study and the SPSS syntax code for statistical analysis are available in the supplementary materials. The SPSS software itself is a commercial product owned by IBM, and its source code is not provided.

## Acknowledgments

This research was supported by a grant from STI2030-Major Projects (2022ZD0211700).

## Author contributions

X.-Y.H. and X.-H.L. conceived the study. J.Z. and Q.-G.Z. supervised the experiments and data analysis. M.-J.X. wrote the manuscript. X.-H.L. and X.-Y.H. performed the experiments and data analysis. J.Z. and Q.-G.Z. provided technical support.

## Declaration of interests

The authors declare no competing interests.

## Supplemental information

Document S1. Representative SPSS output and interpretation for repeated-measures ANOVA of Morris water maze data, related to Step 31-35.

Data S1. Raw water maze data for spatial working memory assessment in mice, related to step 27 and 36a

Data S2. Raw water maze data for reference memory assessment in mice, related to step 36b
