# Supplementary material for: Protocol for the standardized design of Morris water maze experiments and statistical analysis using SPSS
Source: STAR Protoc. 2026 Apr 29;7(2):104509. doi: 10.1016/j.xpro.2026.104509 (PMC13141749; doi:10.1016/j.xpro.2026.104509)

A

Within-Subjects Factors

Measure: MEASURE_1

Day	Dependent Variable
1	Day1
2	Day2
3	Day3
4	Day4
5	Day5

Between-Subjects Factors

		Value Label	N
group	1	1	15
	2	2	15
	3	3	15

Descriptive Statistics

	group	Mean	Std. Deviation	N
Day1	1	42.5833	6.30523	15
	2	43.5833	7.19788	15
	3	40.9793	8.20347	15
	Total	42.3820	7.19192	45
Day2	1	34.8667	10.07776	15
	2	41.9333	10.75435	15
	3	36.2893	2.80829	15
	Total	37.6964	9.00825	45
Day3	1	29.9500	10.14326	15
	2	39.4667	10.34186	15
	3	35.0667	9.01078	15
	Total	34.8278	10.39560	45
Day4	1	26.5667	9.65867	15
	2	43.1833	8.00193	15
	3	33.8213	6.56033	15
	Total	34.5238	10.53886	45
Day5	1	23.5000	8.63754	15
	2	38.8000	10.33769	15
	3	31.1787	3.51822	15
	Total	31.1596	10.07881	45

B

Multivariate Tests[a]

Effect		Value	F	Hypothesis df	Error df	Sig.
Day	Pillai's Trace	.569	12.848[b]	4.000	39.000	<.001
	Wilks' Lambda	.431	12.848[b]	4.000	39.000	<.001
	Hotelling's Trace	1.318	12.848[b]	4.000	39.000	<.001
	Roy's Largest Root	1.318	12.848[b]	4.000	39.000	<.001
Day * group	Pillai's Trace	.345	2.084	8.000	80.000	.047
	Wilks' Lambda	.658	2.272[b]	8.000	78.000	.031
	Hotelling's Trace	.516	2.453	8.000	76.000	.020
	Roy's Largest Root	.509	5.086[c]	4.000	40.000	.002

a. Design: Intercept + group
Within Subjects Design: Day

b. Exact statistic

c. The statistic is an upper bound on F that yields a lower bound on the significance level.

Mauchly's Test of Sphericity[a]

Measure: MEASURE_1

					Epsilon[b]		
Within Subjects Effect	Mauchly's W	Approx. Chi-Square	df	Sig.	Greenhouse-Geisser	Huynh-Feldt	Lower-bound
Day	.810	8.533	9	.482	.904	1.000	.250

Tests the null hypothesis that the error covariance matrix of the orthonormalized transformed dependent variables is proportional to an identity matrix.

a. Design: Intercept + group
Within Subjects Design: Day

b. May be used to adjust the degrees of freedom for the averaged tests of significance. Corrected tests are displayed in the Tests of Within-Subjects Effects table.

C

Tests of Within-Subjects Effects

Measure: MEASURE_1

Source		Type III Sum of Squares	df	Mean Square	F	Sig.
Day	Sphericity Assumed	3173.471	4	793.368	11.630	<.001
	Greenhouse-Geisser	3173.471	3.615	877.956	11.630	<.001
	Huynh-Feldt	3173.471	4.000	793.368	11.630	<.001
	Lower-bound	3173.471	1.000	3173.471	11.630	.001
Day * group	Sphericity Assumed	1265.994	8	158.249	2.320	.022
	Greenhouse-Geisser	1265.994	7.229	175.122	2.320	.027
	Huynh-Feldt	1265.994	8.000	158.249	2.320	.022
	Lower-bound	1265.994	2.000	632.997	2.320	.111
Error(Day)	Sphericity Assumed	11460.162	168	68.215		
	Greenhouse-Geisser	11460.162	151.814	75.488		
	Huynh-Feldt	11460.162	168.000	68.215		
	Lower-bound	11460.162	42.000	272.861		

D

Tests of Within-Subjects Contrasts

Measure: MEASURE_1

Source	Day	Type III Sum of Squares	df	Mean Square	F	Sig.
Day	Linear	2953.166	1	2953.166	46.531	<.001
	Quadratic	87.160	1	87.160	1.365	.249
	Cubic	107.038	1	107.038	2.325	.135
	Order 4	26.107	1	26.107	.262	.611
Day * group	Linear	1119.894	2	559.947	8.823	<.001
	Quadratic	56.900	2	28.450	.445	.644
	Cubic	17.280	2	8.640	.188	.830
	Order 4	71.920	2	35.960	.361	.699
Error(Day)	Linear	2665.577	42	63.466		
	Quadratic	2682.537	42	63.870		
	Cubic	1933.189	42	46.028		
	Order 4	4178.859	42	99.497		

E

Tests of Between-Subjects Effects

Measure: MEASURE_1
Transformed Variable: Average

Source	Type III Sum of Squares	df	Mean Square	F	Sig.
Intercept	293513.288	1	293513.288	3513.324	<.001
group	3723.030	2	1861.515	22.282	<.001
Error	3508.802	42	83.543		

F

Day

Estimates

Measure: MEASURE_1

			95% Confidence Interval	
Day	Mean	Std. Error	Lower Bound	Upper Bound
1	42.382	1.085	40.193	44.571
2	37.696	1.291	35.091	40.302
3	34.828	1.468	31.865	37.791
4	34.524	1.218	32.065	36.982
5	31.160	1.198	28.741	33.578

G

Pairwise Comparisons

Measure: MEASURE_1

					95% Confidence Interval for Difference[b]	
(I) Day	(J) Day	Mean Difference (I-J)	Std. Error	Sig.[b]	Lower Bound	Upper Bound
1	2	4.686*	1.460	.003	1.739	7.632
	3	7.554*	1.762	<.001	3.999	11.110
	4	7.858*	1.610	<.001	4.610	11.107
	5	11.222*	1.605	<.001	7.983	14.462
2	1	-4.686*	1.460	.003	-7.632	-1.739
	3	2.869	1.957	.150	-1.081	6.819
	4	3.173*	1.513	.042	.119	6.226
	5	6.537*	1.829	<.001	2.846	10.227
3	1	-7.554*	1.762	<.001	-11.110	-3.999
	2	-2.869	1.957	.150	-6.819	1.081
	4	.304	2.005	.880	-3.742	4.350
	5	3.668	1.838	.053	-.042	7.378
4	1	-7.858*	1.610	<.001	-11.107	-4.610
	2	-3.173*	1.513	.042	-6.226	-.119
	3	-.304	2.005	.880	-4.350	3.742
	5	3.364	1.747	.061	-.161	6.889
5	1	-11.222*	1.605	<.001	-14.462	-7.983
	2	-6.537*	1.829	<.001	-10.227	-2.846
	3	-3.668	1.838	.053	-7.378	.042
	4	-3.364	1.747	.061	-6.889	.161

Based on estimated marginal means

*. The mean difference is significant at the .05 level.

b. Adjustment for multiple comparisons: Least Significant Difference (equivalent to no adjustments).

H

Multiple Comparisons

Measure: MEASURE_1
LSD

					95% Confidence Interval	
(I) group	(J) group	Mean Difference (I-J)	Std. Error	Sig.	Lower Bound	Upper Bound
1	2	-9.9000*	1.49259	<.001	-12.9122	-6.8878
	3	-3.9737*	1.49259	.011	-6.9859	-.9616
2	1	9.9000*	1.49259	<.001	6.8878	12.9122
	3	5.9263*	1.49259	<.001	2.9141	8.9384
3	1	3.9737*	1.49259	.011	.9616	6.9859
	2	-5.9263*	1.49259	<.001	-8.9384	-2.9141

Based on observed means.
The error term is Mean Square(Error) = 16.709.

*. The mean difference is significant at the .05 level.

I

Multivariate Tests

	Value	F	Hypothesis df	Error df	Sig.
Pillai's trace	.569	12.848[a]	4.000	39.000	<.001
Wilks' lambda	.431	12.848[a]	4.000	39.000	<.001
Hotelling's trace	1.318	12.848[a]	4.000	39.000	<.001
Roy's largest root	1.318	12.848[a]	4.000	39.000	<.001

Each F tests the multivariate effect of Day. These tests are based on the linearly independent pairwise comparisons among the estimated marginal means.

a. Exact statistic

J

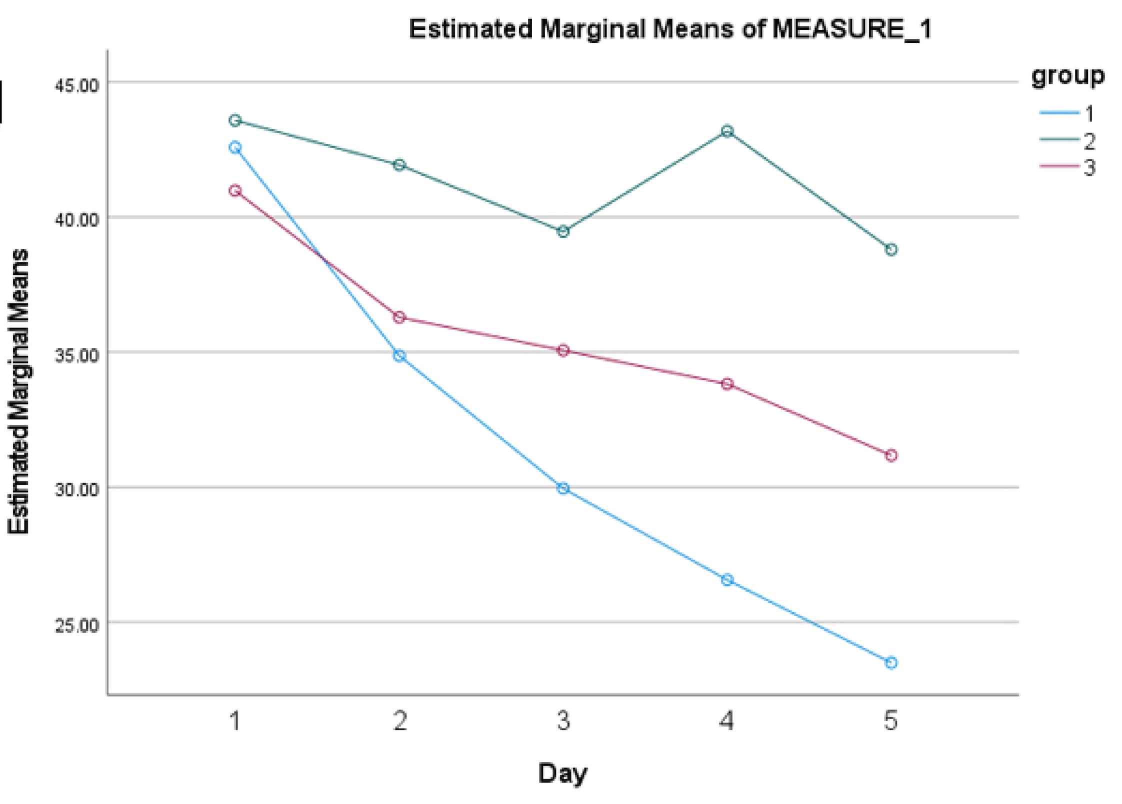

Supplement: Document S1. Representative SPSS output and interpretation for repeated-measures ANOVA of Morris water maze data, related to Step 31-35. [file mmc1.pdf]
